# Electrospinning of Ethylene Vinyl Acetate/Poly(Lactic Acid) Blends on a Water Surface

**DOI:** 10.3390/ma11091737

**Published:** 2018-09-15

**Authors:** Eliška Číková, Jaroslav Kuliček, Ivica Janigová, Mária Omastová

**Affiliations:** Polymer Institute, Slovak Academy of Sciences, Dúbravská cesta 9, 845 41 Bratislava, Slovakia; Eliska.cikova@savba.sk (E.Č.); jaroslav.kulicek@savba.sk (J.K.); ivica.janigova@savba.sk (I.J.)

**Keywords:** electrospinning, ethylene vinyl acetate, poly(lactic acid), blends, properties

## Abstract

The electrospinning of an ethylene vinyl acetate (EVA) copolymer with a vinyl acetate content of 28 wt.% is limited due to the solubility of the copolymer in standard laboratory conditions. Poly(lactic acid) (PLA) is a biodegradable polymer that can be electrospun easily. However, PLA has limited applicability because it is brittle. Blends of these polymers are of interest in order to obtain new types of materials with counterbalanced properties originating from both polymeric compounds. The fibers were electrospun on a water surface from a solution mixture containing various weight ratios of both polymers using a dichloromethane and acetone (70:30 *v*/*v*) mixture as solvent. The morphologies of the prepared non-woven mats were examined by scanning electron microscopy (SEM), and the chemical composition was investigated by X-ray photoelectron spectroscopy (XPS) and by Fourier Transform Infrared Spectroscopy (FTIR). The fibers’ thermal properties and stability were examined, and the mechanical properties were tested. The results showed that the strength and flexibility of the blend samples were enhanced by the presence of PLA.

## 1. Introduction

Ethylene vinyl acetate (EVA) is a random copolymer, consisting of ethylene and vinyl acetate (VAc) units. While the polyethylene units are partially crystalline and stiff, the amorphous vinyl acetate units are flexible and soft. EVA is commercially available, with VAc content varying from 3 to 50 wt.% [1]. The VAc content has two major effects that influence the properties of EVA copolymers; disruption of the crystalline regions formed by the polyethylene segments of the copolymer and the overriding effect of VAc content resulting from the polar nature of the acetoxy side chain [2]. EVA copolymers are transparent, flexible materials with high tensile strength. The disadvantages of EVA copolymers are a reduced chemical resistance compared to LDPE, and reduced barrier properties and creep resistance [1]. EVA copolymers are mainly used in packaging and adhesive applications. Recently, an EVA copolymer was tested as a material for drug release because it is also biocompatible [3,4].

Poly(lactic acid) (PLA) is aliphatic polyester. PLA can be prepared from renewable resources and is biodegradable and biocompatible [5]. PLA has good mechanical properties, such as high strength and stiffness. However, the main drawback of using PLA for most applications is its poor toughness, manifested in its impact strength and elongation at break properties. Under laboratory conditions, PLA is a glassy polymer and is highly brittle. In the food industry, applications of PLA include buffering agents, acidic flavoring agents, acidulates, and bacterial inhibitors [6]. PLA has low melt strength and viscosity, making it difficult to apply in certain processing techniques.

One possibility for improving the mechanical properties of PLA is blending it with other polymers. This method is widely applied due to its feasibility in industry. A number of polymers have been blended with PLA, including various poly(hydroxyalkanoate)s [7,8] and poly(ε-caprolactone) (PCL) [9]. EVA/PLA blends in the form of films have been prepared by melt mixing in an extruder followed by blowing. Two series of PLA/EVA (80/20) were prepared with two EVA copolymers containing 60 and 80 wt.% of VAc, respectively [10]. Films prepared with EVA containing a higher amounts of VAc, showed better overall properties in comparison with films where EVA with 60 wt.% of VAc was mixed. Moura et al. [11] prepared EVA/PLA blends compatibilized with EVA-PLA grafted copolymers by reactive extrusion using a transesterification reaction between EVA and PLA chains with titanium propoxide. Prepared blends were exposed to different environments for characterization of photodurability and biodegradability. Physical blends of PLA with high vinyl acetate containing EVA (50 wt.%) were prepared by melt mixing with 5 to 30 wt.% of EVA and their rheological, thermo-mechanical, and morphological properties were evaluated [12]. 

Electrospinning is a multipurpose and a very popular technique that can generate polymeric fibers, with diameters ranging from less than 100 nm to several μm by creating a continuous filament [13]. This technique depends on various parameters, such as solution properties (e.g., viscosity, surface tension, and conductivity) and processing parameters, e.g., electric field strength, solution flow rate, needle diameter, etc. Thus, by controlling those parameters, well-defined fibers of pure polymer, polymeric blends, or composites can be produced for desirable applications. PLA with varying concentrations of biotin nanofibers was electrospun, and their properties were compared with poly(lactic acid)-block-poly(ethylene glycol) containing biotin for their potential use as biosensors [14]. Nanomaterials based on EVA can be interesting for the industry because the amount of the vinyl acetate can easily change the polarity of the polymer. To the best of our knowledge, there is only one paper that reports electrospinning of various types of EVA with low contents of VAc (28 wt.%, 15 wt.%, and 9 wt.% VAc content); however, not nanofibers but fibers with diameters from 4 to 10 μm were prepared [15]. Authors also reported a change in the melting point of the copolymer and the simultaneous improvement of its mechanical properties. Electrospun fiber mats have been explored as drug delivery vehicles using tetracycline hydrochloride as a model drug. The mats were made either from PLA, EVA (40 wt.% VAc), or from a 50:50 blend of these two polymers. The fibers were electrospun from chloroform solutions containing a small amount of methanol to solubilize the drug [16]. Different percentile combinations of electrospun PCL and EVA (40 wt.% VAc) have been designed for controlled release of tetracycline (tet) HCl from a three-layered electrospun matrix. Electrospinning provides a good encapsulation efficiency of (tet) HCl within polycaprolactone (PCL)/EVA/PCL polymers in micro/nanofiber layers that display sustained antibiotic release [17]. Polymer fibers spun from two-phase suspensions of EVA (40 mol % VAc) dissolved in dichloromethane (DCM) and bovine serum albumin (BSA) dissolved in phosphate-buffered saline can be used for microencapsulation of water droplets (EVA-DCM/BSA-water) [18]. Various types of EVA (28 wt.% VAc) were electrospun with clays [19] or organoclays impregnated with Fe(CO)_5_ [20] to prepare nanocomposites.

In this work, pure EVA and PLA nonwoven mats, as well as blends of both polymers with various ratios were prepared by electrospinning. Only by controlling the temperature of the polymer solution inside the syringe, EVA with a lower amount of VAc content, as has been reported previously, can be prepared by electrospinning. Four polymeric blends with the weight ratios of EVA/PLA = 80:20, 60:40, 40:60, and 20:80 were electrospun. The morphology of the prepared nonwoven mats was investigated and the fibers diameter was determined. X-ray photoelectron spectroscopy (XPS) and Fourier Transform Infrared Spectroscopy (FTIR) were used to study the structure and composition of the prepared fibers. The mechanical and thermal properties of the samples were also investigated. 

## 2. Materials and Methods 

### 2.1. Materials

A commercial type of ethylene–vinyl acetate copolymer (EVA, EVATANE 28–25, Arkema, France) containing 28 wt.% vinyl acetate and poly(lactic acid) (PLA, 4042 D, NatureWorks, Minnetonka, MN, USA) with Mw = 1.65×10^3^ g mol^−1^ were used without further purification. Two solvents, acetone p.a. and dichloromethane p.a. (DCM), were supplied by CentralChem, Slovakia. Distilled water was used as a collector for fibers.

### 2.2. Preparation of Electrospun Fibers

**Figure 1:**
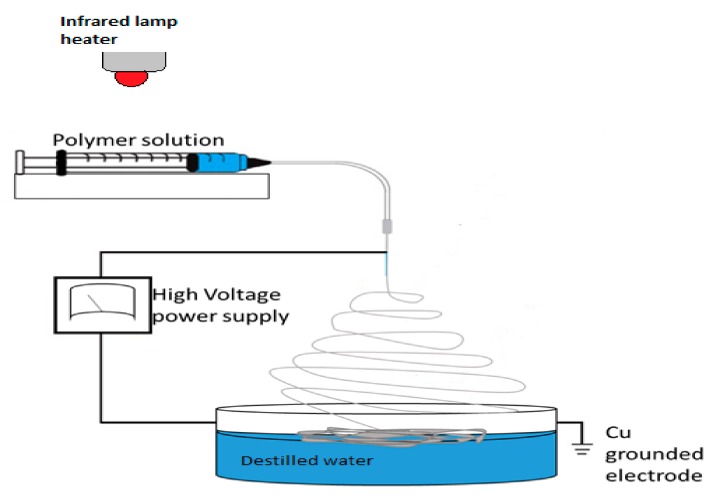
Electrospinning setup.

The 12 wt.% solutions in the solvent mixture were prepared by dissolving various weight ratios of EVA and PLA granules (100:0, 80:20, 60:40, 40:60, 20:80, 0:100) in DCM. According to a previous report [21], acetone was added for the enhanced conductivity of the solutions. The DCM/acetone ratio was set at 70:30 *v*/*v*. The solutions were intensively stirred on a magnetic plate for few hours. Polymers solutions were electrospun from a 5 mL syringe with a metallic needle at the flow rate of 1.5 mL/h. The electrospinning parameters were fixed. A high voltage of 14–15 kV was applied, and the fibers were collected on a distilled water surface containing a copper counter electrode at a distance of 15 cm from the needle tip. The EVA copolymer is not highly soluble at laboratory temperature; therefore, an infrared lamp (Figure 1) was used to heat the polymer solution, as previously described [15,22]. The temperature was controlled by a thermocouple and adjusted to 37 °C. 

### 2.3. Characterization of Prepared Fibers

The scanning electron microscope (SEM) used was a JSM Jeol 6610 microscope (Jeol Ltd., Tokyo, Japan), an accelerated voltage of 10 kV was used for checking the morphology of the electrospun fibers. The samples were sputtered with a thin layer of gold. AzTec software was used for the collection of the micrographs. The images were post-processed using the ImageJ software. ImageJ was also used to measure the average diameter of the fibers in the nonwoven fabrics (the program is available on https://imagej.nih.gov/ij/). For each sample, 100 measurements were randomly made to determine the mean nanofiber diameter.

The samples were analyzed using Fourier Transform Infrared Spectroscopy (FTIR) with a NICOLET 8700™ spectrophotometer (Thermo Scientific, Madison, WI, USA) and an ATR (Attenuated Total Reflection) accessory. The FTIR spectra were analyzed using the OMNIC™ 8.1 software. The analyzed surface was approximately 3.14 mm^2^ (the contact area of the Ge crystal, which was used for ATR measurements), and used range of 4000 to 600 cm^−1^. Three measurements were used for each sample at three different locations. 

Thermo Scientific K-Alpha XPS system (Thermo Fisher Scientific, East Grinstead, UK) equipped with a micro-focused, monochromatic Al Kα X-ray source (1486.6 eV) was used to collect X-ray photoelectron spectra (XPS) of electrospun fibers and also pure EVA and PLA pellets. The constant analyzer energy mode with the pass energy of 200 eV was applied for obtaining the survey scan, narrow regions were collected with the pass energy of 50 eV. For the charge compensation, a flood gun was used. For digital acquisition and data processing, Thermo Scientific Avantage software was used (version 5.988, Thermo Fisher Scientific, East Grinstead, UK). 

Mechanical properties were measured at room temperature using the universal testing machine Instron 3365 (Instron, High Wycombe, UK) using a rate of deformation of 50 mm min^−1^. Seven specimens were tested for each sample, and the final values of tensile strength and elongation at break were averaged. 

The thermal degradation of the samples was investigated using a Mettler-Toledo DSC 821 (Mettler Toledo GmbH, Greifensee, Switzerland) differential scanning calorimeter (DSC). The heat of fusion calibration standard was indium. A temperature interval from −20 to 220 °C was used for samples characterization with a heating rate of 10 °C min^−1^ in airflow. The data were collected during the first heating run, and transition temperatures were taken as peak maxima. Mettler-Toledo 851e thermogravimetric analyzer (Mettler Toledo GmbH, Greifensee, Switzerland) was used for thermogravimetric analysis (TGA). Analysis was carried out in airflow of 50 cm^3^ min^−1^ at the heating rate of 5 °C min^−1^. For both methods, the data were evaluated as average value taken from three samples with SD.

## 3. Results and Discussion

On the basis of our former work, where the EVA copolymer with 28 wt.% VAc was used for the preparation of composites with carbon nanotubes [23] for actuators, we wanted to design nanofibers of EVA and later incorporated carbon-based fillers. As was found in the first experiment, the electrospinning of pure EVA with vinyl acetate content 28 wt.% is highly difficult in standard laboratory conditions. Solutions have to be continuously heated during the electrospinning process to stay in liquid form. The electrospun EVA fibers diameter was in the range of micrometers. Various changes in the experimental conditions (e.g., polymer concentration in solution and voltage) did not provide EVA fibers in the nanometer scale. One of the solutions for preparing nanofibers is the electrospinning of a blend with another polymer, e.g., biodegradable poly(lactic acid). The other problem with electrospinning of EVA is that the electrospun samples tend to be sticky, and if they are collected on aluminum foil, they are destroyed during the necessary removal process for further characterization. The fibers can be collected on the water surface to ease manipulation of the samples without risk of their destruction. From the first study, where various concentrations of EVA in DCM were prepared and electrospun, 12 wt.% concentration in solution was later used for electrospinning because fibrous mats were not produced at lower concentrations. Either electrosprayed droplets or fibers with morphological defects in the form of beads were formed. Similar results were found when EVA was electrospun with clays [19]. The higher concentrations resulted in fibers with higher diameters. The 70:30 solvent mixture of dichloromethane (DCM) and acetone was finally used. DCM is a suitable solvent for both PLA and EVA because some of the traditional solvents used for EVA (e.g., cyclohexane), tend to be selective for VAc or ethylene segments [24]. However, DCM has a lower conductivity (4.3 × 10^−5^ μS/cm), dielectric constant (ε = 9.1), and boiling point (b.p. = 39.6 °C) than acetone (dielectric constant ε = 20.6, conductivity is 0.2 μS/cm, b.p. = 56 °C) [25]. The addition of acetone to the solvent mixture, as well as its amount, was based on previously published reports [19]. It was found that the diameter of the fibers decreased as the boiling point of the second solvent increased [26]. The effect is caused by slower evaporation of the second solvent, thereby leading to a change in viscoelastic properties, which enhances jet stretching and produces thinner fibers [19]. 

**Figure 2:**
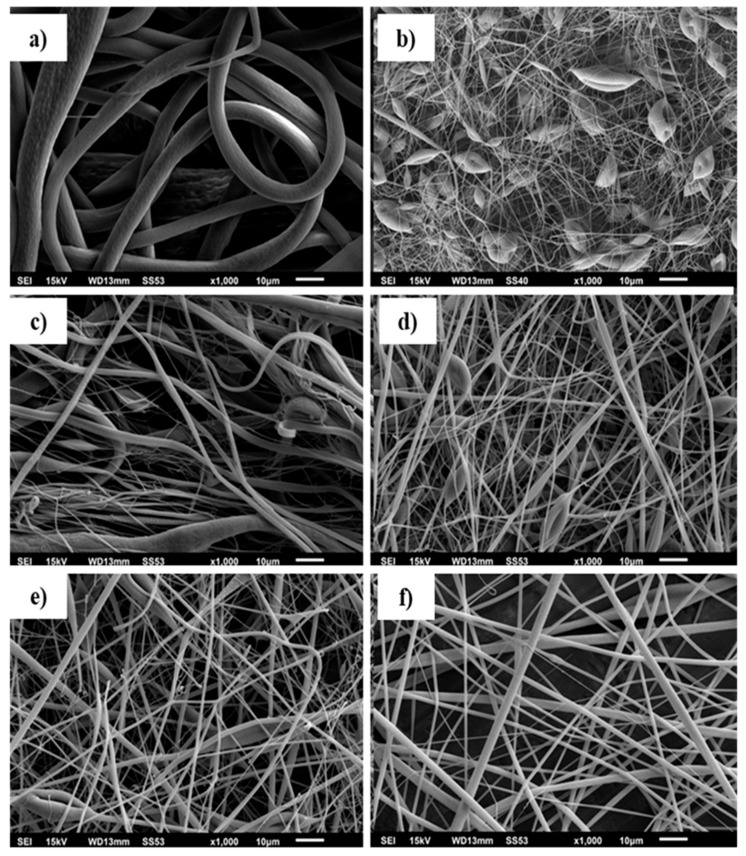
SEM images of the prepared electrospun polymeric mats (**a**) pure EVA, (**b)** pure PLA, (**c**) EVA/PLA 80:20, (**d)**, EVA/PLA 60:40, (**e**) EVA/PLA 40:60, and (**f)** EVA/PLA 20:80.

**Figure 3:**
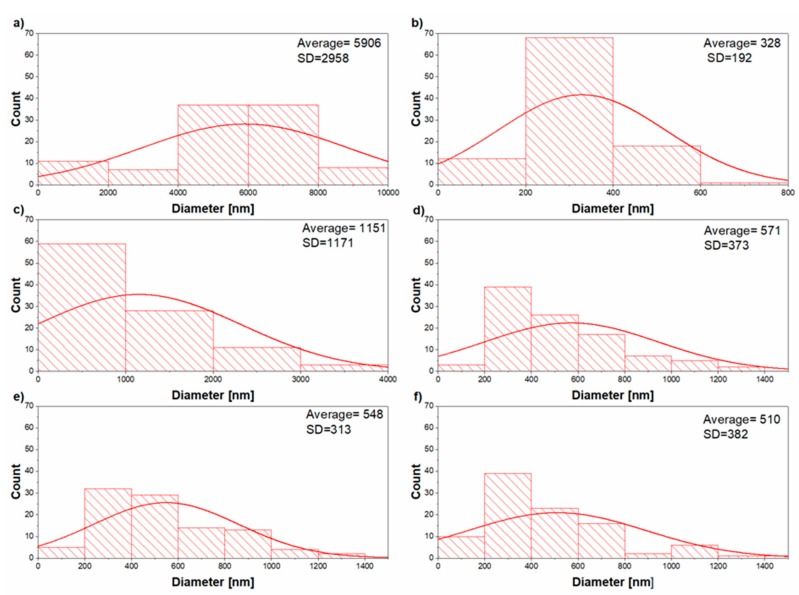
Histogram of the diameter distribution calculated from 100 random fiber measurements: (**a**) pure EVA, (**b**) pure PLA, (**c**) EVA/PLA 80:20, (**d**) EVA/PLA 60:40, (**e**) EVA/PLA 40:60, and (**f**) EVA/PLA 20:80, average value in nm, and standard deviation (SD).

In all of the experiments, the electrospinning conditions were constant, as described in the previous section. Therefore, the study of the electrospun fibrous mats will be discussed regarding EVA to PLA ratios. SEM images of the samples prepared by electrospinning are presented in Figure 2. The solutions of EVA do not have good conductivity, since EVA has low conductivity [1]. This property resulted in fibers with a high diameter (5906 ± 2958 nm). As the content of EVA in the electrospinning solutions of EVA/PLA decreased the diameter of prepared fibers decreased accordingly, but the diameter distribution remained wide (Figure 3). Partial separation of polymers during electrospinning caused a wide distribution of the diameter, resulting in heterogeneous fibers containing blended fibers, as well as EVA and PLA fibers. The bad miscibility of EVA and PLA produced these outcomes. Their miscibility depends on the VAc content. The higher the VAc content (>40 wt.%) is, the better the resulting miscibility is [7,10]. The lower the VAc content is, the higher the interfacial tension is [7]. 

The results are supported by XPS measurements discussed later (Figure 6e). PLA fibers produced from a solvent mixture of DCM/acetone have broad diameter distributions (328 ± 192 nm) with structural defects in the form of beads (Figure 2b). This result is caused by lower conductivity of the PLA solution compared to that of the solvents [21]. In certain cases of electrospun fibers from EVA [15] and low-density poly(ethylene) (LLDPE) [22], rough angular formations were observed. The rapid crystallization process caused these formations during electrospinning. However, similar structures were not found, and the prepared fiber surface was smooth. From Figure 2a–f, it can be noted that the prepared fibers are randomly oriented. Histograms of the diameter distribution calculated from the diameters of 100 random fiber measurements from all the prepared samples are depicted in Figure 3. The SEM image was divided into 8 equal areas, and the diameters of the fibers were measured using ImageJ software. The average diameter of the blends fibers EVA/PLA 60:40, EVA/PLA 40:60, and EVA/PLA 20:80 (Figure 3d,e,f) is nearly the same and reached values from 571 to 510 nm with a notably high standard deviation (SD). The blends EVA/PLA 60:40 and 40:60 contained some beads (Figure 2d,e); however, fibers produced from the EVA/PLA 20:80 mixture are without these defects. 

**Figure 4:**
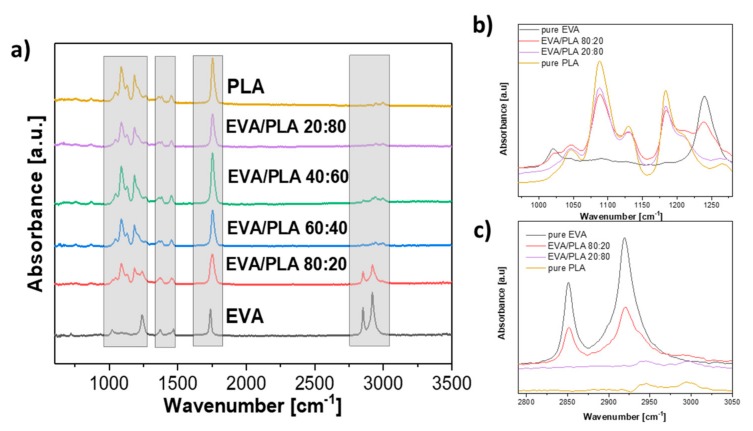
(**a**) FTIR spectra of the prepared mats samples of pure EVA, EVA/PLA 80:20, EVA/PLA 60:40, EVA/PLA 40:60, EVA/PLA, and pure PLA. Details of selected samples spectra (**b**) 970–1280 cm^−1^ and (**c**) 2790–3050 cm^−1^.

Figure 4a shows the ATR FT-IR spectra of the prepared mats and the spectrum of pure electrospun EVA, as well as the spectrum of PLA. Two peaks, which appeared near 2920 cm^−1^ and 2850 cm^−1^, are mainly stretching vibrations of CH and CH_3_ groups [5,27]. Details of selected samples spectra in wavenumbers from 970 to 1280 cm^−1^ and from 2790 to 3050 cm^−1^ are shown in Figure 4b,c respectively. The peak near 1735 cm^−1^ belongs to the carbonyl C=O stretching from both vinyl acetate and PLA, respectively [12]. The intense peak from C–O stretching near 1182 cm^−1^ belongs to PLA [20]. The slightly visible peak near 1453 cm^−1^ is assigned to the bending vibrations of CH_3_ groups [9]. The absorption peak observed at 1366 cm^−1^ is attributed to C–H deformation, including asymmetric and symmetric bending [19]. The peak at 1240 cm^−1^ resp. 1268 cm^−1^ (Figure 4b) is assigned to –C=O bending from EVA and PLA [19,20]. The intense peaks at 1186 and 1087 cm^−1^ belong to C–O stretching from PLA (Figure 4b) [20]. In the FTIR spectra of the electrospun samples, no new peaks or bands appeared. The resemblance between the spectra of the electrospun polymers and blends is noticeable at the first look, which is not surprising, and a similar trend was reported by Moura et al. [28].

**Figure 5:**
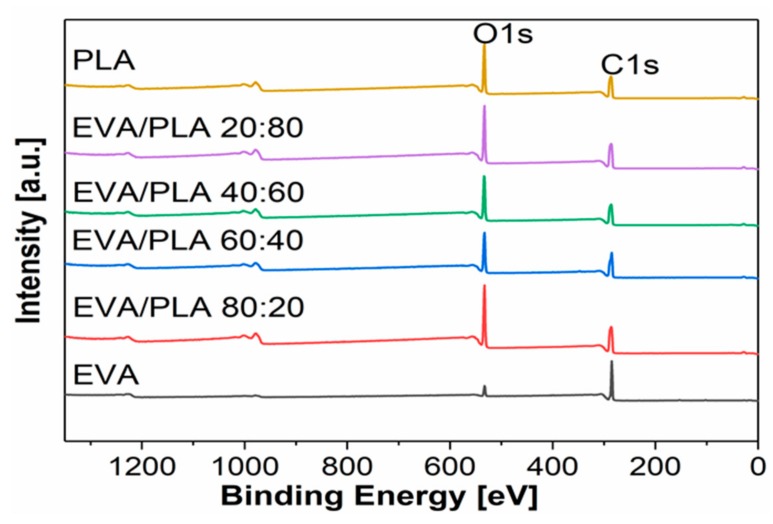
XPS survey spectra of prepared mats samples of pure EVA, EVA/PLA 80:20, EVA/PLA 60:40, EVA/PLA 40:60, EVA/PLA 20:80, and pure PLA.

An XPS study was conducted in order to evaluate the surface of prepared samples (Figure 5). XPS is a surface-sensitive quantitative spectroscopic technique that provides information about the elemental composition and chemical state of investigated samples up to 10 nm depth. Table 1 summarizes the elemental composition of the sample surfaces. Nitrogen N1s (centered at ~399.5 eV), carbon C1s (at ~285 eV), and oxygen O1s (at ~532 eV) signals were detected. The small amount of detected nitrogen is possibly from the ambient air during the preparation and collection of the fibers on the water surface. The high resolution C1 peaks were deconvoluted and fitted with five components. O1s peaks were fitted with two components, C=O and C–O, centered at 532.1 eV and 533.4 eV, respectively.

**Figure 6:**
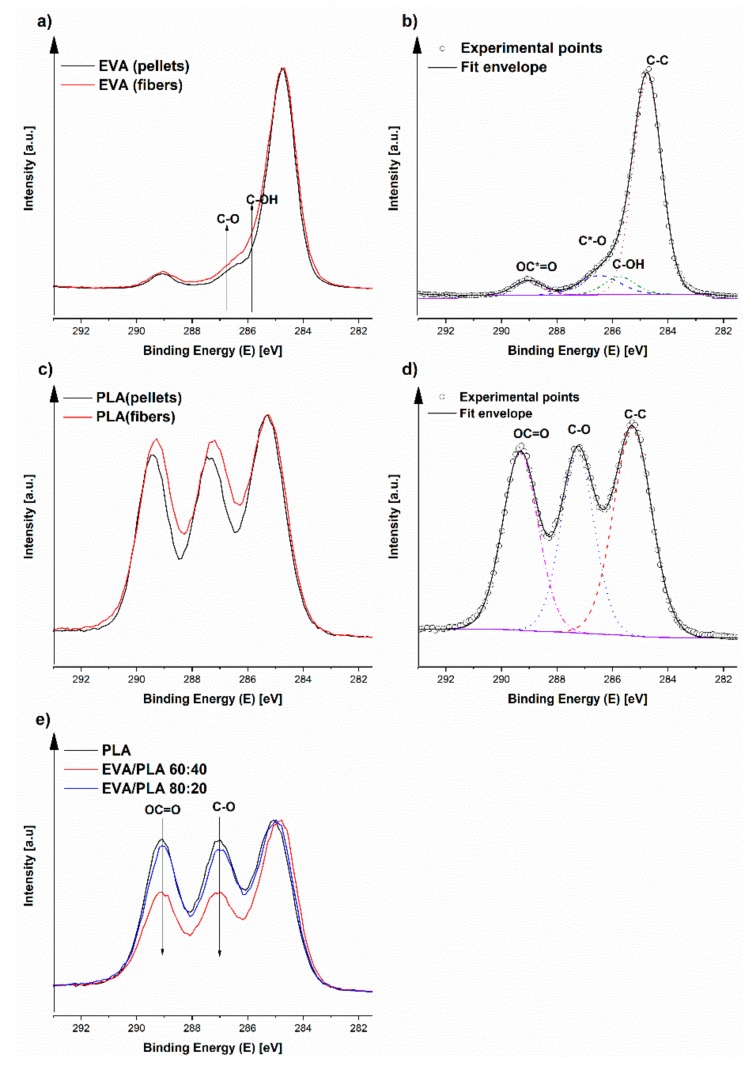
Comparison of C1s peaks of selected samples and their deconvolution.

The first step of the XPS investigation was comparison of the chemical composition for the pure polymers prepared by electrospinning with the original samples in the form of pellets. In the original EVA pellets, 90.1 at.% of C and 9.9 at.% of O was detected. EVA typically has a higher carbon content which is connected to the higher amount of ethylene (C–C signal at ~285 eV) compared to vinyl acetate, which contains carboxyl groups OC=O (C1s centered at ~289 eV, O1s (C=O) centered at ~532 eV) [29]. Electrospun EVA contains more oxygen, 12.4 at.%., when compared to EVA pellets, which indicates oxidation during the fiber processing. This oxidation most likely occurs on the polyethylene chain of EVA, which confirms the increase of signal at a 285.8 eV corresponding to a C–O/C–OH signal (see Figure 6a,b) [29]. From the comparison of the composition of EVA and PLA, PLA contains more oxygen. The oxygen content is connected to the polymer structure containing a C–O group (C1s centered at ~286 eV, O1s centered at ~533 eV) and OC=O group (C1s centered at ~289 eV and O1s (C=O) centered at ~532 eV), which is typical for PLA [29]. In the PLA pellets, 62.8 at.% of C and 37.0 at.% oxygen was found. The chemical composition of electrospun PLA fibers is highly similar, as only a small amount of nitrogen was found in this sample. In the case of PLA, there was no change in oxygen content before and after electrospinning. After electrospinning, the ratio of C–C/C–O/OC=O is closer to the stoichiometry of PLA, which could be attributed to the sample being less contaminated by adventitious carbon in the case of electrospun PLA (Table 1, and Figure 6c,d). However, after electrospinning, the signals of C–O (~286 eV) and OC=O (~289 eV) appear a slightly broader, which indicates more variations of the carbon–oxygen species, and although the overall oxygen has not increased, a degree of degradation/oxidation of PLA occurred. In this case, we have not attempted to deconvolute some of the additional signals, as in the case of EVA. This finding is observed because the signals are almost at the same positions and are only slightly broader. From the elemental composition in Table 1, it is obvious that the three EVA/PLA samples prepared from different ratios of both polymers have a similar composition to pure PLA (C1s, O1s), only one, EVA/PLA 60:40, is different. This finding indicates that PLA dominates on the surface in the other three EVA/PLA fiber mats.

From the results obtained in Table 1, significant difference in the surface composition of the EVA/PLA 60:40 sample was found compared with the results from the other samples. Figure 6e shows a comparison of C1s peaks of PLA and EVA/PLA fibers prepared with the ratios of 60:40 and 80:20. In the case of EVA/PLA 80:20 comparison to pure PLA, pure PLA is more visible, which signifies that PLA is dominant on the surface. There is no change in oxygen content, and only a small decrease in the C–O/OC=O portion is indicated in Figure 6e. In the case of EVA/PLA 60:40, the contribution of the polyethylene chain from EVA (strongest signal at ca. 285 eV corresponding to C–C relative to C–O/OC=O signals) is clearly observed, which confirms EVA is on the surface. 

**Figure 7:**
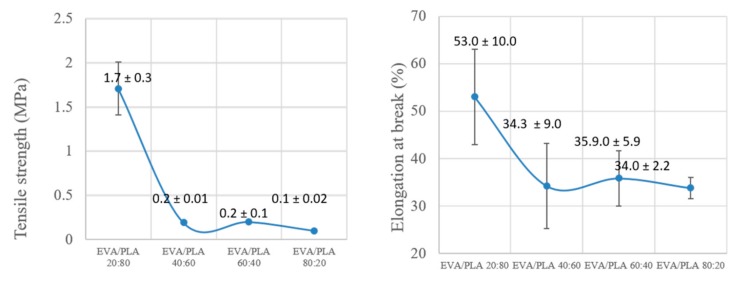
Effect of EVA/PLA ratio on tensile strength (**a**) and elongation at break (**b**) of prepared fibrous mats.

The mechanical properties of a prepared material is essential data for its application. EVA is a flexible elastomer; however, it is not easily electrospun. The mechanical testing of pure electrospun EVA was not possible due to the infirmity of the sample. The weakness of the electrospun EVA compared to the blended EVA/PLA could be caused by partial degradation on the ethylene groups as revealed by XPS. It is also known that PLA is a brittle polymer when it is prepared in the form of a film or electrospun mats. The brittleness of PLA, compared to the blend and the presence of beads in the sample (Figure 2), leads to an impossibility for mechanical testing. From the literature, it is known that with a combination of EVA with PLA at a ratio 80:20, it is possible to create a tough blend [7]. In this case, samples in the form of films were investigated. It can be explained as strong interfacial adhesion between EVA and PLA [30] caused by the elastomeric nature of EVA. The enhanced mechanical properties due to the higher content of PLA can be explained as enhancing the flexibility of PLA by EVA, while reducing the tensile values of PLA (Figure 7). In these cases, the flexible EVA acts as an energy absorber during deformation and leads to the higher toughness of the samples. The samples with higher content of EVA are almost two times weaker with tensile stress around 0.15 MPa than the sample EVA/PLA 20:80 (1.71 ± 0.3 MPa) and have significantly lower flexibility. The tensile strain of EVA/PLA 20:80 was 53.0 ± 10.1% compared to other samples with values close to 35%. This result is caused by the miscibility of the polymers and with a lower amount of PLA, a decreased the number of chain entanglements and covalent bonds [10] are observed, as confirmed by SEM (Figure 2c–e and Figure 3c–e ) and XPS (Figure 6e). The tensile properties of the prepared electrospun EVA/PLA sheet are enhanced with increasing PLA content.

**Figure 8:**
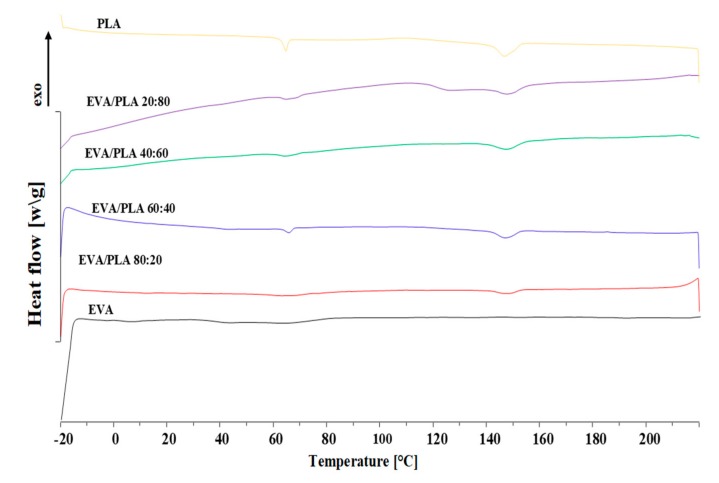
DSC thermographs of the first heating for pure EVA, PLA, and EVA/PLA blends.

Thermal behaviors of the electrospun non-woven mats were investigated by TGA and DSC analysis. The results of the DSC investigation of PLA and electrospun blends are shown in Figure 8. The essential transition temperatures, glass transition temperature (T*_g_*), and melting point (T*_m_*) were determined from the first heating, and the cold crystallization temperature (T*_cc_*) from cooling. All the results obtained are summarized in Table 2. The T*_g_* of the pure PLA fibers was determined to be 61.4 °C. As it can be seen from measured data, the amount of EVA present in the blend samples influenced the blends T*_g_*. With increasing the EVA amount in the blends, the shifting of T_g_ to higher temperatures was achieved. The T*_g_* of EVA/PLA blends containing 20 wt.% of EVA is 62.8 °C, while for blend with the inverse ratio, T*_g_* = 66.0 °C was determined. The T_m_ of pure PLA is 146.7 °C. According to the EVA datasheet, melting temperature of this copolymer is in the range of 60 to 90 °C; therefore, the presence of EVA in blends did not significantly change the melting temperature. The small change of T*_g_* and T*_m_* is caused by low miscibility of the samples with higher concentrations of EVA. The molecular chains in electrospun fibers are highly oriented and aligned, and this behavior is erased in the cooling process, during which the crystallization is finished [31]. The weak cold crystallization peak corresponds to the slow crystallization rate of PLA. For EVA/PLA 80:20, T*_cc_* was assigned to 92.2 °C. 

The T*_cc_* peak is shifted to higher temperatures with increased PLA content. The difference of T*_cc_* between blend samples is almost 15 °C. 

**Figure 9:**
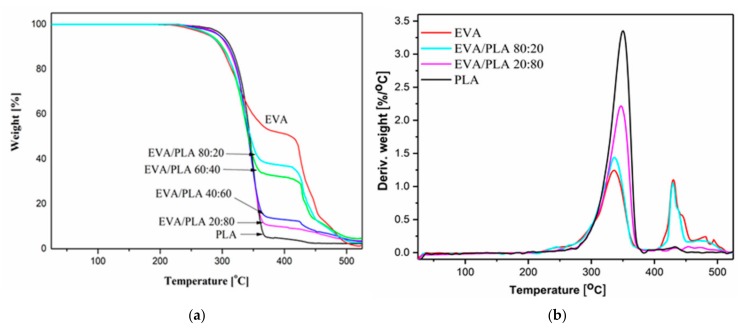
TGA curves of pure EVA, PLA, and blended EVA/PLA samples (**a**), and DTG curves for pure EVA, PLA, and selected blended samples (**b**).

Figure 9 shows TGA curves of neat EVA, PLA, and EVA/PLA mats. The degradation temperatures at 10 and 50% of mass losses for all the studied samples and maximum decomposition temperature (T_max_) were determined from derivation of the obtained TGA curves (selected DTG curves are shown in Figure 9b). 

The thermal degradation of EVA is a two-step process. The first part is a loss of vinyl acetate content in the form of acetic acid [32] followed by degradation of the unsaturated polyethylene [33,34]. For the studied EVA fibers, first T1_max_ was located at 334.5 ± 0.8 °C and T2_max_ at 431.2 ± 1.5 °C. Pure PLA degrades in a single step with the main peak centered approximately 270–385 °C. This degradation is due to the intramolecular transesterification reaction [30]. For the electrospun PLA fibers, the maximum decomposition temperature was determined at 351.4 ± 0.3 °C. The electrospun blends showed a two-stage decomposition process. The first step is in the range of 250 to 389 °C and corresponds to PLA degradation. The T1_max_ for the first degradation step is from 322.0 ± 2.0 °C for the sample with highest EVA content to 345.0 ± 0.4 °C for blend with lower EVA content. The T2_max_ for the blends fibers is very near to the T2_max_ of the pure EVA. The final stage of decomposition is the degradation of polyethylene (400–520 °C). For the EVA/PLA 20:80 blends fibers, the second decomposition maximum was not clearly detected. The addition of PLA into the blends leads to a less intense second peak. The degradation temperatures of all the prepared samples have been compared at two points, 10 and 50% mass losses, and the results are shown in Table 3. There is a visible stabilizing effect caused by the presence of PLA, with an increasing amount of PLA, T_10%_ is shifted from 301 to 316 °C. The T_50%_ is influenced by presence of PLA, and almost the same temperature, approximately 345 °C, was determined for blended fibers samples.

## 4. Conclusions

Electrospinning of EVA copolymer with 28 wt.% vinyl acetate content in laboratory conditions is possible only when the solution is continuously heated, but the prepared fiber’s diameter was in the range of 6 microns. To obtain fibrous mats with fiber diameters in the nanometer range, EVA was blended with polylactic acid (PLA) in a DCM/acetone solution using various weight ratios of EVA/PLA: 80:20, 60:40, 40:60, and 20:80. Pure PLA fibers mats were also prepared for comparison. The properties of the electrospun EVA/PLA blends depend on the polymers ratios. Morphology studies revealed the presence of small portions of beads in blended samples. Only fibers produced from a blend of EVA/PLA 20:80 were without these defects and reached a diameter of approximately 510 nm, which is ten times less than the diameter of the prepared EVA fibers. The XPS study showed that electrospun EVA contained more oxygen compared to EVA pellets, which indicates a slight oxidation during the fiber processing. The XPS also detected that EVA/PLA blends contained PLA on the surface. Only at the 60:40 ratio was EVA detected in the outer fibers. The prepared blends, however, showed better mechanical properties than pure PLA and reached better thermo-oxidation stability than EVA at a temperature of 10% mass loss. The electrospun EVA/PLA 20:80 blend ideally combined the properties of both polymers, which resulted in better mechanical properties than the other samples. The obtained results showed that electrospinning was a suitable method for producing EVA/PLA blends, which can be potentially used in various biomedical applications. This will be a topic for future study. 

## Figures and Tables

**Table 1:** Elemental surface chemical composition of the prepared blends mats and EVA and PLA pellets for comparison with C1s, and O1s deconvoluted peaks fitting parameters (in at.%).

Sample	Surface Chemical Composition [at.%]		
	C1s C-C//C-OH//C-O//C=O//OC=O *(284.8//285.7//286.3//286.9//289.2)*	O1s C=O//C-O *(532.1/533.4)*	N1s NC=O *(399.5)*
EVA (pellets)	90.1 *84.6//-//8.2//2.0//5.2*	9.9 *49.5//50.5*	—
EVA	87.5 *77.3//7.1//9.9//-//5.6*	12.4 *50.9//49.1*	0.1 *100.0*
EVA/PLA 80:20	63.1 *39.4//-//29.2//-//31.5*	36.9 *39.4//60.6*	—
EVA/PLA 60:40	63.9 *47.5//-//26.3//-//26.4*	35.7 *45.0//55.0*	0.3 *100.0*
EVA/PLA 40:60	63.8 *38.3//-//25.6//-//36.1*	36.0 *26.3//73.7*	0.2 *100.0*
EVA/PLA 20:80	63.6 *39.2//-//29.0//-//31.8*	36.3 *45.0//55.0*	0.1 *100.0*
PLA	62.7 *39.3//-//29.6//-//31.1*	37.0 *38.8//61.2*	0.3 *100.0*
PLA (pellets)	63.0 *41.6//-//29.4//-//28.9*	37.0 *44.7//55.3*	—

**Table 2:** DSC analysis of EVA/PLA blends and pure EVA and PLA electrospun samples from the first heating.

Sample	T*_g_* [°C]	T*_cc_* [°C]	T*_m_* [°C]
EVA	-	-	66.4 ± 0.4
EVA/PLA 80:20	66.2 ± 0.3	92.8 ± 1.5	148.1 ± 0.3
EVA/PLA 60:40	63.7 ± 0.1	110.0 ± 1.0	147.3 ± 0.1
EVA/PLA 40:60	63.7 ± 1.0	111.1 ± 1.7	147.8 ± 0.1
EVA/PLA 20:80	62.5 ± 0.3	107.0 ± 1.0	148.2 ± 0.4
PLA	62.0 ± 0.4	109.8 ± 1.0	146.8 ± 0.6

**Table 3:** Degradation temperatures at 10 and 50% mass losses, and maximum decomposition temperatures for the first and the second degradation step of prepared samples.

Sample	T10% [°C]	T50% [°C]	First Degradation Step			Second Degradation Step		
			T1onset [°C]	T1max [°C]	T1f [°C]	T2onset [°C]	T2max [°C]	T2f [°C]
EVA	297.8 ± 0.9	411.8 ± 1.0	259.8 ± 0.6	334.5 ± 0.8	387.6 ± 1.0	405.6 ± 2.5	431.2 ± 1.5	457.4 ± 0.7
EVA/PLA 80:20	301.2 ± 0.4	345.2 ± 0.7	264.0 ± 1.1	322.0 ± 2.0	387.1 ± 2.2	411.0 ± 0.7	428.2 ± 1.8	453.3 ± 3.0
EVA/PLA 60:40	304.0 ± 1.0	343.0 ± 0.2	273.9 ± 0.2	341.2 ± 0.4	388.9 ± 1.9	414.5 ± 1.0	425.9 ± 0.7	440.8 ± 0.8
EVA/PLA 40:60	312.8 ± 0.7	343.2 ± 0.5	274.3 ± 0.7	342.2 ± 1.2	386.7 ± 1.2	416.7 ± 1.5	426.7 ± 2.1	442.1 ± 1.3
EVA/PLA 20:80	313.3 ± 0.6	343.3 ± 0.7	274.2 ± 0.9	345.0 ± 0.4	392.6 ± 0.8	-	-	-
PLA	315.9 ± 0.2	344.4 ± 0.4	275.7 ± 0.8	351.4 ± 0.3	384.7 ± 1.4	-	-	-
